# 4-{(*E*)-2-[4-(Diethyl­amino)­phen­yl]ethen­yl}-1-methyl­pyridin-1-ium tetra­phenyl­borate

**DOI:** 10.1107/S1600536812034575

**Published:** 2012-08-11

**Authors:** Dan-Dan Li, Rui Li, Sheng-Li Li

**Affiliations:** a Deparment of Chemistry, Anhui University, Hefei 230039, People’s Republic of China; b Key Laboratory of Functional Inorganic Materials, Chemistry, Hefei 230039, People’s Republic of China

## Abstract

In the cation of the title salt, C_18_H_23_N_2_^+^·C_24_H_20_B^−^, the pyridine ring forms a dihedral angle of 14.23 (6)° with the benzene ring. One of the ethyl groups of the cation was refined as disordered over two sets of sites with equal occupancies.

## Related literature

For the use of stilbazolium compounds as non-linear optical materials, see: Hao *et al.* (2009 ▶); Zhou *et al.* (2011 ▶). For the crystal structure of a related pyridinium derivative, see: Li *et al.* (2000 ▶).
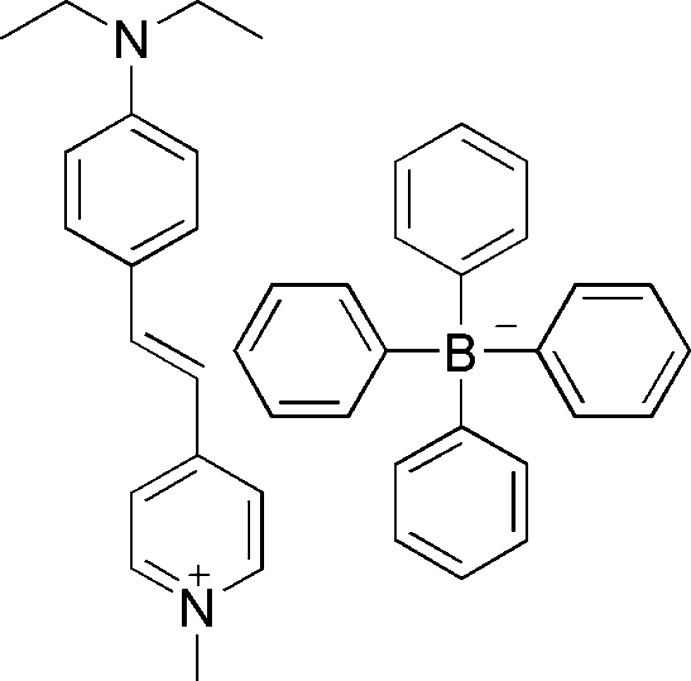


## Experimental

### 

#### Crystal data

C_18_H_23_N_2_^+^·C_24_H_20_B^−^
*M* *_r_* = 586.59
Monoclinic, 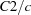

*a* = 38.59 (8) Å
*b* = 11.35 (2) Å
*c* = 17.04 (3) Å
β = 101.43 (5)°
*V* = 7314 (25) Å^3^
*Z* = 8
Mo *K*α radiation
μ = 0.06 mm^−1^
*T* = 298 K
0.30 × 0.20 × 0.20 mm

#### Data collection

Bruker SMART CCD diffractometer
Absorption correction: multi-scan (SABADS; Bruker, 2007 ▶) *T*_min_ = 0.982, *T*_max_ = 0.988
24610 measured reflections
6411 independent reflections
4032 reflections with *I* > 2σ(*I*)
*R*_int_ = 0.038

#### Refinement

*R*[*F* ^2^ > 2σ(*F* ^2^)] = 0.056
*wR*(*F* ^2^) = 0.199
*S* = 1.03
6411 reflections
429 parameters
H-atom parameters constrained
Δρ_max_ = 0.38 e Å^−3^
Δρ_min_ = −0.18 e Å^−3^

### 

Data collection: *SMART* (Bruker, 2007 ▶); cell refinement: *SAINT* (Bruker, 2007 ▶); data reduction: *SAINT*; program(s) used to solve structure: *SHELXS97* (Sheldrick, 2008 ▶); program(s) used to refine structure: *SHELXL97* (Sheldrick, 2008 ▶); molecular graphics: *SHELXTL* (Sheldrick, 2008 ▶); software used to prepare material for publication: *SHELXTL* (Sheldrick, 2008 ▶).

## Supplementary Material

Crystal structure: contains datablock(s) I, global. DOI: 10.1107/S1600536812034575/lh5509sup1.cif

Structure factors: contains datablock(s) I. DOI: 10.1107/S1600536812034575/lh5509Isup2.hkl

Additional supplementary materials:  crystallographic information; 3D view; checkCIF report

## Figures and Tables

**Fig. 1.**
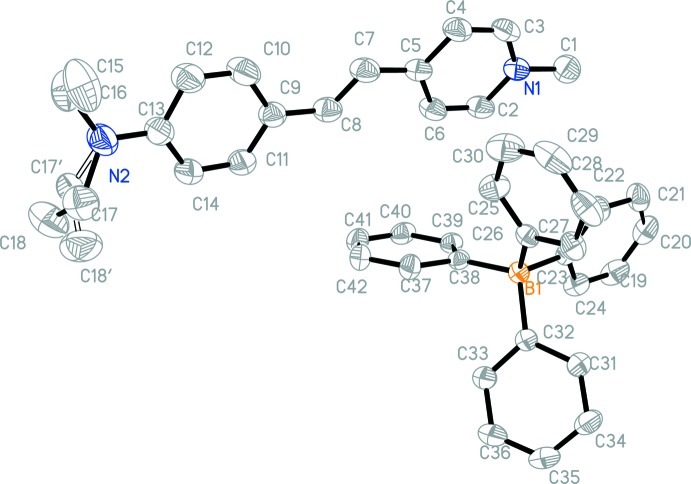
: The molecular structure of the title compound (I) showing 30% probability displacement ellipsoids.

## supplementary crystallographic information

### Comment

The use of stilbazolium compounds as nonlinear optical materials is a newly emerging field that has already exhibited potential applications in upconverted lasing and biological imaging because of their high stability and tailor-ability (Hao *et al.*, 2009; Zhou *et al.*, 2011). The importance of the structures of pyridinium derivatives has already been reported (Li *et al.*, 2000). We have synthesized title compound and it's crystal structure is presented herein. The asymmetric unit of the title compound (I) is shown in Fig.1. In the cation, the pyridine ring makes a dihedral angle of 14.23 (6) ° with the benzene ring and the C═C bond is 1.353 (4) Å. While in a similar cation (Li *et al.*, 2000), the corresponding value of the dihedral angle is 19.94 (4) ° and the C═C bond is 1.226 (4) Å. This may indicate that the π-electron delocalization in the title cation is enhanced compared to the literature structure. This feature is a necessary condition for the cation to bear a large two-photon absorption (TPA) cross-section.

### Experimental

A 100 mL round-buttom flask was charged with a magnetic stirrer and a reflux condenser, 1.77 g (0.01 mol) of 4-(*N*,*N*-diethylamino)-benzaldehyde, 2.36 g (0.01 mol) of 1,4-dimethylpyridinium iodide and 30 mL of absolute ethanol and mixed. Five drops of piperidine were added into the mixture. The solution was then heated to reflux for 4 h. The solution was kept warm and filtered into an ethanol solution of sodium tetraphenylborate (3.46 g, 0.01 mol) with stirring. A red precipitate appeared, the solution was filtered and the solid was washed with ethanol three times and dried over vacuum. ^1^H NMR: (400 Hz, CD_3_COCD_3_), d(p.p.m.): 8.62(d, 2H) 8.04(d, 2H) 7.89(d, 1H) 7.60(d, 2H) 7.35(s, 8H) 7.15(d, 1H) 6.94–6.91(t, 8H) 6.79–6.77(q, 6H) 4.35(s, 3H) 3.53–3.48(q, 4H) 1.22–1.18(t, 6H). Single crystals were obtained from slow evaporation over several days of an acetonitrile solution of the title compound at room temperature.

### Refinement

All hydrogen atoms were placed in geometrically idealized positions and constrained to ride on their parent atoms, with C—H = 0.93–0.97 Å, *U*_iso_(H) = 1.2 *U*_eq_(C) or 1.5*U*_eq_(C). Atoms C17 and C18 atoms of an ethyl group were refined as disordered over two sets of sites (C17/C17',C18/C18') with the equall occupancies.

### Crystal data

**Table:**

C_18_H_23_N_2_^+^·C_24_H_20_B^−^	*F*(000) = 2512
*M**_r_* = 586.59	*D*_x_ = 1.065 Mg m^−^^3^
Monoclinic, *C*2/*c*	Mo *K*α radiation, λ = 0.71073 Å
Hall symbol: -C 2yc	Cell parameters from 4692 reflections
*a* = 38.59 (8) Å	θ = 2.2–21.1°
*b* = 11.35 (2) Å	µ = 0.06 mm^−^^1^
*c* = 17.04 (3) Å	*T* = 298 K
β = 101.43 (5)°	Block, red
*V* = 7314 (25) Å^3^	0.30 × 0.20 × 0.20 mm
*Z* = 8	

### Data collection

**Table:**

Bruker SMART CCD diffractometer	6411 independent reflections
Radiation source: fine-focus sealed tube	4032 reflections with *I* > 2σ(*I*)
Graphite monochromator	*R*_int_ = 0.038
φ and ω scans	θ_max_ = 25.0°, θ_min_ = 1.1°
Absorption correction: multi-scan (SABADS; Bruker, 2007)	*h* = −45→45
*T*_min_ = 0.982, *T*_max_ = 0.988	*k* = −13→13
24610 measured reflections	*l* = −19→20

### Refinement

**Table:**

Refinement on *F*^2^	Secondary atom site location: difference Fourier map
Least-squares matrix: full	Hydrogen site location: inferred from neighbouring sites
*R*[*F*^2^ > 2σ(*F*^2^)] = 0.056	H-atom parameters constrained
*wR*(*F*^2^) = 0.199	*w* = 1/[σ^2^(*F*_o_^2^) + (0.1058*P*)^2^ + 2.2113*P*] where *P* = (*F*_o_^2^ + 2*F*_c_^2^)/3
*S* = 1.03	(Δ/σ)_max_ < 0.001
6411 reflections	Δρ_max_ = 0.38 e Å^−^^3^
429 parameters	Δρ_min_ = −0.18 e Å^−^^3^
0 restraints	Extinction correction: *SHELXL97* (Sheldrick, 2008), Fc^*^=kFc[1+0.001xFc^2^λ^3^/sin(2θ)]^-1/4^
Primary atom site location: structure-invariant direct methods	Extinction coefficient: 0.0026 (4)

### Special details

**Table:**

Geometry. All e.s.d.'s (except the e.s.d. in the dihedral angle between two l.s. planes) are estimated using the full covariance matrix. The cell e.s.d.'s are taken into account individually in the estimation of e.s.d.'s in distances, angles and torsion angles; correlations between e.s.d.'s in cell parameters are only used when they are defined by crystal symmetry. An approximate (isotropic) treatment of cell e.s.d.'s is used for estimating e.s.d.'s involving l.s. planes.
Refinement. Refinement of *F*^2^ against ALL reflections. The weighted *R*-factor *wR* and goodness of fit *S* are based on *F*^2^, conventional *R*-factors *R* are based on *F*, with *F* set to zero for negative *F*^2^. The threshold expression of *F*^2^ > σ(*F*^2^) is used only for calculating *R*-factors(gt) *etc*. and is not relevant to the choice of reflections for refinement. *R*-factors based on *F*^2^ are statistically about twice as large as those based on *F*, and *R*- factors based on ALL data will be even larger.

### Fractional atomic coordinates and isotropic or equivalent isotropic displacement parameters (Å^2^)

**Table:**

	*x*	*y*	*z*	*U*_iso_*/*U*_eq_	Occ. (<1)
C1	0.29481 (8)	0.8332 (3)	−0.11485 (18)	0.0922 (9)	
H1A	0.2851	0.8507	−0.0685	0.138*	
H1B	0.2768	0.7989	−0.1555	0.138*	
H1C	0.3032	0.9045	−0.1349	0.138*	
C2	0.35515 (7)	0.7803 (2)	−0.03925 (15)	0.0774 (7)	
H2	0.3567	0.8558	−0.0177	0.093*	
C3	0.32304 (8)	0.6360 (3)	−0.12374 (16)	0.0856 (8)	
H3	0.3027	0.6131	−0.1595	0.103*	
C4	0.35039 (8)	0.5567 (2)	−0.10350 (17)	0.0850 (8)	
H4	0.3482	0.4820	−0.1264	0.102*	
C5	0.38180 (7)	0.5866 (2)	−0.04856 (14)	0.0709 (7)	
C6	0.38307 (7)	0.7036 (2)	−0.01714 (15)	0.0774 (7)	
H6	0.4031	0.7284	0.0189	0.093*	
C7	0.41081 (8)	0.4997 (2)	−0.02579 (16)	0.0827 (8)	
H7	0.4086	0.4281	−0.0528	0.099*	
C8	0.44025 (7)	0.5163 (2)	0.03111 (16)	0.0794 (7)	
H8	0.4422	0.5896	0.0559	0.095*	
C9	0.46976 (7)	0.4341 (2)	0.05928 (16)	0.0762 (7)	
C10	0.47528 (9)	0.3258 (3)	0.02145 (19)	0.1020 (10)	
H10	0.4594	0.3040	−0.0247	0.122*	
C11	0.49640 (8)	0.4644 (3)	0.12557 (18)	0.0949 (9)	
H11	0.4952	0.5376	0.1493	0.114*	
C12	0.50366 (9)	0.2509 (3)	0.0509 (2)	0.1056 (10)	
H12	0.5065	0.1822	0.0231	0.127*	
C13	0.52850 (8)	0.2777 (3)	0.12316 (18)	0.0884 (8)	
C14	0.52439 (8)	0.3898 (3)	0.15717 (18)	0.0999 (10)	
H14	0.5408	0.4142	0.2017	0.120*	
C15	0.56172 (10)	0.0850 (3)	0.1121 (3)	0.1197 (12)	
H15A	0.5577	0.0974	0.0547	0.144*	
H15B	0.5859	0.0581	0.1298	0.144*	
C16	0.53722 (11)	−0.0051 (4)	0.1305 (3)	0.1550 (18)	
H16A	0.5433	−0.0250	0.1863	0.233*	
H16B	0.5388	−0.0742	0.0989	0.233*	
H16C	0.5135	0.0249	0.1182	0.233*	
C17	0.5724 (3)	0.2030 (8)	0.2461 (8)	0.109 (3)	0.50
H17A	0.5560	0.2393	0.2753	0.130*	0.50
H17B	0.5773	0.1233	0.2659	0.130*	0.50
C18	0.6061 (3)	0.2736 (8)	0.2563 (8)	0.139 (4)	0.50
H18A	0.6228	0.2328	0.2312	0.209*	0.50
H18B	0.6158	0.2834	0.3123	0.209*	0.50
H18C	0.6011	0.3495	0.2317	0.209*	0.50
B1	0.34219 (6)	0.77574 (19)	0.22741 (14)	0.0468 (5)	
C19	0.29162 (9)	1.0842 (2)	0.16706 (17)	0.0832 (8)	
H19	0.2936	1.1610	0.1864	0.100*	
C20	0.26494 (8)	1.0558 (3)	0.10160 (18)	0.0845 (9)	
H20	0.2488	1.1126	0.0779	0.101*	
C21	0.26295 (7)	0.9405 (3)	0.07249 (16)	0.0798 (8)	
H21	0.2457	0.9205	0.0283	0.096*	
C22	0.28725 (6)	0.8532 (2)	0.11015 (14)	0.0638 (6)	
H22	0.2854	0.7773	0.0892	0.077*	
C23	0.31413 (5)	0.87685 (17)	0.17827 (12)	0.0501 (5)	
C25	0.33930 (7)	0.5731 (2)	0.13919 (16)	0.0762 (7)	
H25	0.3594	0.6000	0.1222	0.091*	
C26	0.32598 (5)	0.64056 (17)	0.19768 (12)	0.0507 (5)	
C27	0.29573 (6)	0.5909 (2)	0.22178 (15)	0.0672 (6)	
H27	0.2863	0.6298	0.2608	0.081*	
C28	0.27940 (8)	0.4855 (2)	0.18919 (19)	0.0871 (9)	
H28	0.2595	0.4574	0.2065	0.105*	
C29	0.29290 (9)	0.4229 (2)	0.1309 (2)	0.0944 (10)	
H29	0.2821	0.3538	0.1092	0.113*	
C30	0.32320 (10)	0.4665 (2)	0.10577 (19)	0.0977 (10)	
H30	0.3326	0.4258	0.0675	0.117*	
C31	0.31225 (6)	0.8110 (2)	0.35908 (14)	0.0685 (6)	
H31	0.2905	0.8052	0.3240	0.082*	
C32	0.34388 (5)	0.79782 (16)	0.32660 (12)	0.0486 (5)	
C33	0.37542 (6)	0.8111 (2)	0.38470 (13)	0.0660 (6)	
H33	0.3969	0.8049	0.3682	0.079*	
C34	0.31223 (8)	0.8322 (2)	0.44106 (16)	0.0788 (7)	
H34	0.2909	0.8383	0.4585	0.095*	
C35	0.34419 (8)	0.8441 (2)	0.49558 (15)	0.0793 (8)	
H35	0.3445	0.8587	0.5494	0.095*	
C36	0.37584 (8)	0.8334 (3)	0.46701 (15)	0.0863 (8)	
H36	0.3974	0.8411	0.5025	0.104*	
C37	0.41046 (6)	0.7081 (2)	0.23451 (14)	0.0640 (6)	
H37	0.4054	0.6445	0.2647	0.077*	
C38	0.38276 (5)	0.79148 (17)	0.20609 (12)	0.0491 (5)	
C39	0.39338 (6)	0.88629 (18)	0.16085 (12)	0.0556 (5)	
H39	0.3769	0.9444	0.1415	0.067*	
C40	0.42829 (7)	0.8960 (2)	0.14391 (14)	0.0690 (7)	
H40	0.4338	0.9586	0.1133	0.083*	
C41	0.45417 (7)	0.8112 (3)	0.17342 (16)	0.0786 (8)	
H41	0.4768	0.8170	0.1629	0.094*	
C42	0.44500 (6)	0.7175 (2)	0.21912 (16)	0.0749 (7)	
H42	0.4619	0.6611	0.2395	0.090*	
C17'	0.5865 (3)	0.2476 (10)	0.2149 (5)	0.104 (2)	0.50
H17C	0.5906	0.3296	0.2034	0.125*	0.50
H17D	0.6077	0.2034	0.2120	0.125*	0.50
C18'	0.5784 (4)	0.2371 (11)	0.3013 (6)	0.152 (4)	0.50
H18D	0.5604	0.2927	0.3074	0.228*	0.50
H18E	0.5995	0.2534	0.3401	0.228*	0.50
H18F	0.5704	0.1587	0.3094	0.228*	0.50
N1	0.32500 (5)	0.74767 (19)	−0.09248 (12)	0.0713 (6)	
N2	0.55643 (8)	0.2019 (2)	0.15412 (17)	0.1149 (10)	
C24	0.31557 (7)	0.99717 (19)	0.20423 (14)	0.0670 (6)	
H24	0.3331	1.0190	0.2475	0.080*	

### Atomic displacement parameters (Å^2^)

**Table:**

	*U*^11^	*U*^22^	*U*^33^	*U*^12^	*U*^13^	*U*^23^
C1	0.093 (2)	0.100 (2)	0.0819 (19)	0.0139 (17)	0.0135 (16)	0.0148 (16)
C2	0.0921 (19)	0.0679 (16)	0.0677 (16)	−0.0060 (14)	0.0048 (14)	−0.0120 (12)
C3	0.0823 (19)	0.095 (2)	0.0708 (17)	−0.0091 (16)	−0.0058 (14)	−0.0178 (15)
C4	0.090 (2)	0.0755 (17)	0.0819 (18)	−0.0042 (15)	−0.0005 (15)	−0.0246 (14)
C5	0.0828 (17)	0.0685 (15)	0.0588 (14)	−0.0055 (13)	0.0074 (13)	−0.0088 (12)
C6	0.0829 (18)	0.0726 (17)	0.0691 (16)	−0.0076 (14)	−0.0036 (13)	−0.0100 (13)
C7	0.099 (2)	0.0670 (16)	0.0756 (17)	−0.0034 (14)	0.0022 (16)	−0.0142 (13)
C8	0.0918 (19)	0.0707 (16)	0.0737 (17)	−0.0045 (14)	0.0113 (15)	−0.0114 (13)
C9	0.0857 (18)	0.0695 (16)	0.0704 (16)	0.0048 (14)	0.0085 (14)	−0.0049 (13)
C10	0.120 (2)	0.0785 (19)	0.090 (2)	0.0057 (18)	−0.0204 (18)	−0.0089 (16)
C11	0.098 (2)	0.091 (2)	0.087 (2)	0.0181 (17)	−0.0002 (17)	−0.0305 (16)
C12	0.131 (3)	0.0734 (18)	0.099 (2)	0.0153 (18)	−0.010 (2)	−0.0166 (16)
C13	0.094 (2)	0.0807 (19)	0.0825 (19)	0.0094 (16)	−0.0018 (16)	−0.0072 (15)
C14	0.098 (2)	0.108 (2)	0.083 (2)	0.0186 (18)	−0.0081 (16)	−0.0300 (17)
C15	0.098 (2)	0.104 (3)	0.153 (3)	0.020 (2)	0.016 (2)	0.012 (2)
C16	0.115 (3)	0.142 (3)	0.195 (4)	0.002 (3)	−0.003 (3)	0.069 (3)
C17	0.134 (7)	0.093 (6)	0.099 (8)	0.003 (5)	0.024 (6)	0.023 (5)
C18	0.140 (8)	0.135 (7)	0.123 (8)	−0.023 (6)	−0.023 (7)	0.003 (6)
B1	0.0486 (13)	0.0428 (12)	0.0484 (13)	0.0010 (10)	0.0080 (10)	0.0038 (10)
C19	0.124 (2)	0.0582 (15)	0.0749 (18)	0.0348 (15)	0.0375 (18)	0.0174 (13)
C20	0.098 (2)	0.085 (2)	0.0785 (19)	0.0445 (16)	0.0369 (17)	0.0408 (15)
C21	0.0647 (16)	0.096 (2)	0.0751 (17)	0.0156 (14)	0.0063 (13)	0.0314 (15)
C22	0.0619 (14)	0.0625 (14)	0.0649 (14)	0.0053 (11)	0.0074 (11)	0.0139 (11)
C23	0.0547 (12)	0.0485 (11)	0.0493 (12)	0.0041 (9)	0.0159 (10)	0.0099 (9)
C25	0.0962 (19)	0.0550 (14)	0.0789 (17)	−0.0105 (13)	0.0210 (14)	−0.0106 (12)
C26	0.0545 (12)	0.0434 (11)	0.0499 (12)	0.0041 (9)	−0.0002 (9)	0.0090 (9)
C27	0.0602 (14)	0.0637 (14)	0.0743 (16)	−0.0065 (11)	0.0052 (12)	0.0095 (12)
C28	0.0756 (18)	0.0677 (17)	0.105 (2)	−0.0261 (14)	−0.0145 (16)	0.0215 (16)
C29	0.119 (3)	0.0469 (14)	0.096 (2)	−0.0167 (15)	−0.0298 (19)	0.0119 (15)
C30	0.140 (3)	0.0559 (16)	0.093 (2)	−0.0069 (17)	0.013 (2)	−0.0153 (14)
C31	0.0647 (15)	0.0825 (16)	0.0597 (14)	0.0083 (12)	0.0155 (12)	−0.0005 (12)
C32	0.0561 (12)	0.0394 (10)	0.0505 (12)	0.0042 (9)	0.0111 (10)	0.0059 (8)
C33	0.0608 (14)	0.0801 (16)	0.0567 (14)	−0.0035 (12)	0.0106 (11)	−0.0058 (12)
C34	0.0900 (19)	0.0884 (18)	0.0656 (16)	0.0106 (15)	0.0339 (15)	−0.0018 (13)
C35	0.109 (2)	0.0811 (17)	0.0509 (14)	0.0028 (15)	0.0235 (15)	−0.0057 (12)
C36	0.0862 (19)	0.112 (2)	0.0551 (15)	−0.0046 (16)	0.0001 (14)	−0.0115 (14)
C37	0.0587 (14)	0.0638 (14)	0.0705 (15)	0.0087 (11)	0.0152 (11)	0.0022 (11)
C38	0.0543 (12)	0.0458 (11)	0.0471 (11)	0.0005 (9)	0.0099 (9)	−0.0053 (9)
C39	0.0612 (13)	0.0528 (12)	0.0530 (12)	−0.0058 (10)	0.0118 (10)	−0.0061 (10)
C40	0.0732 (16)	0.0746 (16)	0.0654 (15)	−0.0207 (13)	0.0285 (13)	−0.0107 (12)
C41	0.0543 (15)	0.104 (2)	0.0803 (18)	−0.0088 (14)	0.0207 (13)	−0.0248 (16)
C42	0.0547 (14)	0.0897 (18)	0.0803 (17)	0.0141 (13)	0.0129 (12)	−0.0071 (14)
C17'	0.098 (6)	0.114 (7)	0.089 (6)	0.032 (5)	−0.009 (5)	−0.007 (5)
C18'	0.224 (12)	0.151 (8)	0.081 (6)	0.046 (8)	0.033 (7)	0.013 (6)
N1	0.0780 (14)	0.0769 (14)	0.0572 (12)	−0.0009 (11)	0.0092 (10)	−0.0008 (10)
N2	0.120 (2)	0.0963 (18)	0.109 (2)	0.0327 (16)	−0.0254 (17)	−0.0195 (16)
C24	0.0902 (17)	0.0544 (13)	0.0586 (14)	0.0135 (12)	0.0202 (12)	0.0087 (11)

### Geometric parameters (Å, º)

**Table:**

C1—N1	1.506 (4)	C19—H19	0.9300
C1—H1A	0.9600	C20—C21	1.396 (5)
C1—H1B	0.9600	C20—H20	0.9300
C1—H1C	0.9600	C21—C22	1.426 (4)
C2—N1	1.376 (4)	C21—H21	0.9300
C2—C6	1.378 (4)	C22—C23	1.421 (4)
C2—H2	0.9300	C22—H22	0.9300
C3—N1	1.371 (4)	C23—C24	1.433 (4)
C3—C4	1.378 (4)	C25—C30	1.426 (4)
C3—H3	0.9300	C25—C26	1.430 (4)
C4—C5	1.418 (4)	C25—H25	0.9300
C4—H4	0.9300	C26—C27	1.428 (4)
C5—C6	1.428 (4)	C27—C28	1.413 (4)
C5—C7	1.485 (4)	C27—H27	0.9300
C6—H6	0.9300	C28—C29	1.403 (5)
C7—C8	1.353 (4)	C28—H28	0.9300
C7—H7	0.9300	C29—C30	1.412 (5)
C8—C9	1.476 (4)	C29—H29	0.9300
C8—H8	0.9300	C30—H30	0.9300
C9—C11	1.411 (4)	C31—C34	1.418 (4)
C9—C10	1.424 (4)	C31—C32	1.444 (4)
C10—C12	1.399 (5)	C31—H31	0.9300
C10—H10	0.9300	C32—C33	1.417 (4)
C11—C14	1.394 (4)	C33—C36	1.422 (4)
C11—H11	0.9300	C33—H33	0.9300
C12—C13	1.435 (4)	C34—C35	1.396 (4)
C12—H12	0.9300	C34—H34	0.9300
C13—N2	1.398 (4)	C35—C36	1.407 (5)
C13—C14	1.420 (4)	C35—H35	0.9300
C14—H14	0.9300	C36—H36	0.9300
C15—C16	1.468 (5)	C37—C42	1.413 (4)
C15—N2	1.539 (5)	C37—C38	1.437 (3)
C15—H15A	0.9700	C37—H37	0.9300
C15—H15B	0.9700	C38—C39	1.430 (3)
C16—H16A	0.9600	C39—C40	1.437 (4)
C16—H16B	0.9600	C39—H39	0.9300
C16—H16C	0.9600	C40—C41	1.406 (4)
C17—C18	1.508 (14)	C40—H40	0.9300
C17—N2	1.567 (12)	C41—C42	1.403 (4)
C17—H17A	0.9700	C41—H41	0.9300
C17—H17B	0.9700	C42—H42	0.9300
C18—H18A	0.9600	C17'—N2	1.486 (11)
C18—H18B	0.9600	C17'—C18'	1.569 (14)
C18—H18C	0.9600	C17'—H17C	0.9700
B1—C23	1.682 (4)	C17'—H17D	0.9700
B1—C38	1.686 (4)	C18'—H18D	0.9600
B1—C26	1.696 (4)	C18'—H18E	0.9600
B1—C32	1.697 (4)	C18'—H18F	0.9600
C19—C20	1.397 (5)	C24—H24	0.9300
C19—C24	1.413 (4)		
			
N1—C1—H1A	109.5	C22—C23—C24	114.18 (19)
N1—C1—H1B	109.5	C22—C23—B1	124.9 (2)
H1A—C1—H1B	109.5	C24—C23—B1	121.0 (2)
N1—C1—H1C	109.5	C30—C25—C26	122.9 (3)
H1A—C1—H1C	109.5	C30—C25—H25	118.5
H1B—C1—H1C	109.5	C26—C25—H25	118.5
N1—C2—C6	121.6 (3)	C27—C26—C25	114.6 (2)
N1—C2—H2	119.2	C27—C26—B1	123.03 (19)
C6—C2—H2	119.2	C25—C26—B1	122.0 (2)
N1—C3—C4	121.7 (3)	C28—C27—C26	123.2 (3)
N1—C3—H3	119.1	C28—C27—H27	118.4
C4—C3—H3	119.1	C26—C27—H27	118.4
C3—C4—C5	121.4 (3)	C29—C28—C27	120.5 (3)
C3—C4—H4	119.3	C29—C28—H28	119.7
C5—C4—H4	119.3	C27—C28—H28	119.7
C4—C5—C6	115.5 (2)	C28—C29—C30	118.9 (3)
C4—C5—C7	121.0 (2)	C28—C29—H29	120.6
C6—C5—C7	123.5 (2)	C30—C29—H29	120.6
C2—C6—C5	121.2 (2)	C29—C30—C25	119.8 (3)
C2—C6—H6	119.4	C29—C30—H30	120.1
C5—C6—H6	119.4	C25—C30—H30	120.1
C8—C7—C5	125.3 (3)	C34—C31—C32	124.1 (2)
C8—C7—H7	117.4	C34—C31—H31	117.9
C5—C7—H7	117.4	C32—C31—H31	117.9
C7—C8—C9	129.0 (3)	C33—C32—C31	113.3 (2)
C7—C8—H8	115.5	C33—C32—B1	124.7 (2)
C9—C8—H8	115.5	C31—C32—B1	121.90 (19)
C11—C9—C10	115.0 (3)	C32—C33—C36	123.3 (2)
C11—C9—C8	120.0 (3)	C32—C33—H33	118.4
C10—C9—C8	124.9 (3)	C36—C33—H33	118.4
C12—C10—C9	122.6 (3)	C35—C34—C31	120.0 (3)
C12—C10—H10	118.7	C35—C34—H34	120.0
C9—C10—H10	118.7	C31—C34—H34	120.0
C14—C11—C9	123.2 (3)	C34—C35—C36	118.3 (3)
C14—C11—H11	118.4	C34—C35—H35	120.8
C9—C11—H11	118.4	C36—C35—H35	120.8
C10—C12—C13	121.5 (3)	C35—C36—C33	121.0 (2)
C10—C12—H12	119.3	C35—C36—H36	119.5
C13—C12—H12	119.3	C33—C36—H36	119.5
N2—C13—C14	122.5 (3)	C42—C37—C38	123.6 (2)
N2—C13—C12	121.8 (3)	C42—C37—H37	118.2
C14—C13—C12	115.5 (3)	C38—C37—H37	118.2
C11—C14—C13	121.8 (3)	C39—C38—C37	113.8 (2)
C11—C14—H14	119.1	C39—C38—B1	125.01 (18)
C13—C14—H14	119.1	C37—C38—B1	121.2 (2)
C16—C15—N2	110.5 (4)	C38—C39—C40	123.1 (2)
C16—C15—H15A	109.5	C38—C39—H39	118.4
N2—C15—H15A	109.5	C40—C39—H39	118.4
C16—C15—H15B	109.5	C41—C40—C39	120.2 (2)
N2—C15—H15B	109.5	C41—C40—H40	119.9
H15A—C15—H15B	108.1	C39—C40—H40	119.9
C15—C16—H16A	109.5	C42—C41—C40	118.5 (3)
C15—C16—H16B	109.5	C42—C41—H41	120.7
H16A—C16—H16B	109.5	C40—C41—H41	120.7
C15—C16—H16C	109.5	C41—C42—C37	120.7 (2)
H16A—C16—H16C	109.5	C41—C42—H42	119.6
H16B—C16—H16C	109.5	C37—C42—H42	119.6
C18—C17—N2	106.1 (11)	N2—C17'—C18'	110.8 (13)
C18—C17—H17A	110.5	N2—C17'—H17C	109.5
N2—C17—H17A	110.5	C18'—C17'—H17C	109.5
C18—C17—H17B	110.5	N2—C17'—H17D	109.5
N2—C17—H17B	110.5	C18'—C17'—H17D	109.5
H17A—C17—H17B	108.7	H17C—C17'—H17D	108.1
C23—B1—C38	110.74 (19)	C17'—C18'—H18D	109.5
C23—B1—C26	107.8 (2)	C17'—C18'—H18E	109.5
C38—B1—C26	109.56 (16)	H18D—C18'—H18E	109.5
C23—B1—C32	106.80 (17)	C17'—C18'—H18F	109.5
C38—B1—C32	110.19 (17)	H18D—C18'—H18F	109.5
C26—B1—C32	111.73 (16)	H18E—C18'—H18F	109.5
C20—C19—C24	120.6 (3)	C3—N1—C2	118.6 (2)
C20—C19—H19	119.7	C3—N1—C1	121.1 (2)
C24—C19—H19	119.7	C2—N1—C1	120.3 (2)
C21—C20—C19	118.6 (2)	C13—N2—C17'	118.9 (4)
C21—C20—H20	120.7	C13—N2—C15	121.2 (3)
C19—C20—H20	120.7	C17'—N2—C15	117.9 (4)
C20—C21—C22	120.4 (3)	C13—N2—C17	119.5 (5)
C20—C21—H21	119.8	C15—N2—C17	114.2 (4)
C22—C21—H21	119.8	C19—C24—C23	123.0 (3)
C23—C22—C21	123.0 (2)	C19—C24—H24	118.5
C23—C22—H22	118.5	C23—C24—H24	118.5
C21—C22—H22	118.5		
			
N1—C3—C4—C5	0.7 (5)	C23—B1—C32—C33	−128.5 (2)
C3—C4—C5—C6	−0.8 (4)	C38—B1—C32—C33	−8.2 (3)
C3—C4—C5—C7	178.7 (3)	C26—B1—C32—C33	113.9 (2)
N1—C2—C6—C5	−0.2 (4)	C23—B1—C32—C31	48.2 (2)
C4—C5—C6—C2	0.5 (4)	C38—B1—C32—C31	168.52 (18)
C7—C5—C6—C2	−178.9 (3)	C26—B1—C32—C31	−69.4 (2)
C4—C5—C7—C8	−173.8 (3)	C31—C32—C33—C36	0.9 (3)
C6—C5—C7—C8	5.6 (4)	B1—C32—C33—C36	177.8 (2)
C5—C7—C8—C9	178.0 (3)	C32—C31—C34—C35	1.3 (4)
C7—C8—C9—C11	−173.3 (3)	C31—C34—C35—C36	−0.5 (4)
C7—C8—C9—C10	10.8 (5)	C34—C35—C36—C33	0.0 (4)
C11—C9—C10—C12	4.2 (5)	C32—C33—C36—C35	−0.2 (4)
C8—C9—C10—C12	−179.7 (3)	C42—C37—C38—C39	−0.4 (3)
C10—C9—C11—C14	−5.9 (5)	C42—C37—C38—B1	−179.4 (2)
C8—C9—C11—C14	177.8 (3)	C23—B1—C38—C39	7.4 (3)
C9—C10—C12—C13	1.9 (6)	C26—B1—C38—C39	126.2 (2)
C10—C12—C13—N2	178.4 (3)	C32—B1—C38—C39	−110.5 (2)
C10—C12—C13—C14	−6.4 (5)	C23—B1—C38—C37	−173.75 (18)
C9—C11—C14—C13	1.5 (5)	C26—B1—C38—C37	−55.0 (3)
N2—C13—C14—C11	180.0 (3)	C32—B1—C38—C37	68.3 (2)
C12—C13—C14—C11	4.7 (5)	C37—C38—C39—C40	1.3 (3)
C24—C19—C20—C21	−1.5 (4)	B1—C38—C39—C40	−179.83 (19)
C19—C20—C21—C22	1.3 (4)	C38—C39—C40—C41	−1.2 (3)
C20—C21—C22—C23	0.5 (4)	C39—C40—C41—C42	0.2 (3)
C21—C22—C23—C24	−2.1 (3)	C40—C41—C42—C37	0.6 (4)
C21—C22—C23—B1	177.0 (2)	C38—C37—C42—C41	−0.5 (4)
C38—B1—C23—C22	105.1 (2)	C4—C3—N1—C2	−0.3 (4)
C26—B1—C23—C22	−14.7 (3)	C4—C3—N1—C1	−179.7 (3)
C32—B1—C23—C22	−134.9 (2)	C6—C2—N1—C3	0.1 (4)
C38—B1—C23—C24	−75.7 (3)	C6—C2—N1—C1	179.4 (2)
C26—B1—C23—C24	164.43 (18)	C14—C13—N2—C17'	−11.6 (8)
C32—B1—C23—C24	44.2 (2)	C12—C13—N2—C17'	163.4 (6)
C30—C25—C26—C27	1.6 (3)	C14—C13—N2—C15	−175.3 (3)
C30—C25—C26—B1	−171.7 (2)	C12—C13—N2—C15	−0.4 (5)
C23—B1—C26—C27	−72.8 (3)	C14—C13—N2—C17	31.2 (7)
C38—B1—C26—C27	166.66 (18)	C12—C13—N2—C17	−153.8 (6)
C32—B1—C26—C27	44.3 (2)	C18'—C17'—N2—C13	86.4 (9)
C23—B1—C26—C25	100.0 (3)	C18'—C17'—N2—C15	−109.3 (6)
C38—B1—C26—C25	−20.5 (3)	C18'—C17'—N2—C17	−15.3 (8)
C32—B1—C26—C25	−142.9 (2)	C16—C15—N2—C13	−78.9 (5)
C25—C26—C27—C28	−1.8 (3)	C16—C15—N2—C17'	117.1 (7)
B1—C26—C27—C28	171.5 (2)	C16—C15—N2—C17	75.8 (6)
C26—C27—C28—C29	0.8 (4)	C18—C17—N2—C13	−101.2 (8)
C27—C28—C29—C30	0.4 (4)	C18—C17—N2—C17'	−1.2 (7)
C28—C29—C30—C25	−0.5 (4)	C18—C17—N2—C15	103.6 (7)
C26—C25—C30—C29	−0.6 (4)	C20—C19—C24—C23	−0.3 (4)
C34—C31—C32—C33	−1.5 (3)	C22—C23—C24—C19	2.0 (3)
C34—C31—C32—B1	−178.5 (2)	B1—C23—C24—C19	−177.2 (2)
